# Predicting patient survival after deceased donor kidney transplantation using flexible parametric modelling

**DOI:** 10.1186/s12882-016-0264-0

**Published:** 2016-05-25

**Authors:** Bernadette Li, John A. Cairns, Matthew L. Robb, Rachel J. Johnson, Christopher J. E. Watson, John L. Forsythe, Gabriel C. Oniscu, Rommel Ravanan, Christopher Dudley, Paul Roderick, Wendy Metcalfe, Charles R. Tomson, J. Andrew Bradley

**Affiliations:** Department of Health Services Research and Policy, London School of Hygiene and Tropical Medicine, 15-17 Tavistock Place, London, WC1H 9SH UK; NHS Blood and Transplant, Bristol, UK; Department of Surgery, University of Cambridge and the NIHR Cambridge Biomedical Research Centre, Cambridge, UK; Transplant Unit, Royal Infirmary of Edinburgh, Edinburgh, UK; Richard Bright Renal Unit, Southmead Hospital, Bristol, UK; Primary Care and Population Sciences, Faculty of Medicine, University of Southampton, Southampton, UK; Scottish Renal Registry, Glasgow, UK; Department of Renal Medicine, Freeman Hospital, Newcastle upon Tyne, UK

**Keywords:** Kidney transplantation, Survival, Multivariable analysis, Flexible parametric model, Extrapolation

## Abstract

**Background:**

The influence of donor and recipient factors on outcomes following kidney transplantation is commonly analysed using Cox regression models, but this approach is not useful for predicting long-term survival beyond observed data. We demonstrate the application of a flexible parametric approach to fit a model that can be extrapolated for the purpose of predicting mean patient survival. The primary motivation for this analysis is to develop a predictive model to estimate post-transplant survival based on individual patient characteristics to inform the design of alternative approaches to allocating deceased donor kidneys to those on the transplant waiting list in the United Kingdom.

**Methods:**

We analysed data from over 12,000 recipients of deceased donor kidney or combined kidney and pancreas transplants between 2003 and 2012. We fitted a flexible parametric model incorporating restricted cubic splines to characterise the baseline hazard function and explored a range of covariates including recipient, donor and transplant-related factors.

**Results:**

Multivariable analysis showed the risk of death increased with recipient and donor age, diabetic nephropathy as the recipient’s primary renal diagnosis and donor hypertension. The risk of death was lower in female recipients, patients with polycystic kidney disease and recipients of pre-emptive transplants. The final model was used to extrapolate survival curves in order to calculate mean survival times for patients with specific characteristics.

**Conclusion:**

The use of flexible parametric modelling techniques allowed us to address some of the limitations of both the Cox regression approach and of standard parametric models when the goal is to predict long-term survival.

## Background

Outcomes following kidney transplantation are commonly analysed using Cox regression models. Such analyses have been instrumental for understanding the influence of both donor and recipient factors on post-transplant events, such as graft failure and patient mortality [1–5]. However, the Cox regression approach places emphasis on estimating relative risk and does not make any distributional assumptions about the absolute risk of an event. Therefore, its usefulness in predicting survival beyond the period of observed data is limited [6]. Following kidney transplantation, the risk of death is highest in the period immediately after surgery, but decreases sharply and then changes direction when the risk of death starts to gradually increase over time. While a number of standard parametric models (such as the exponential, Weibull or loglogistic) are available and could facilitate extrapolation of survival data, they are not flexible enough to accommodate hazard functions that change direction.

In some situations, we not only want to understand what factors influence relative survival, but we also want to predict long-term survival for patients with given characteristics. Estimates of life expectancy following transplant are important as a basis for having informed discussions with individual patients and their relatives. For decision-making at a population level, estimates of mean survival are needed to inform cost-effectiveness evaluations that compare two or more treatment alternatives in terms of both lifetime health gains and costs. There has also been considerable interest in the development of survival prediction models and scoring tools for use in kidney allocation systems. A number of predictive models have been proposed, such as the Recipient Risk Score (RRS), Life Years From Transplant (LYFT) and the Expected Post Transplant Survival (EPTS) score, the latter which was adopted as a measure alongside the Kidney Donor Profile Index (KDPI) to facilitate longevity matching in the revised kidney allocation system approved by the Organ Procurement and Transplantation Network in the United States in 2013 [7–11]. The primary motivation for the current analysis is to develop a predictive model to estimate post-transplant survival as a potential approach to inform the design alternative allocation schemes for deceased donor kidneys in the United Kingdom.

In order to estimate mean patient survival from observed data, it is desirable to have complete information about when most or all patients have died. If the data are not complete, estimates of mean survival will not reflect the full distribution of survival times and will likely underestimate true survival [12]. For recipients of kidney transplants, waiting to observe post-transplant mortality for a complete cohort of patients would require several decades of follow-up. To circumvent this problem, predictive models such as the aforementioned LYFT approach used estimates of median rather than mean survival times [8]. In contrast to the mean, median survival only requires sufficient follow-up to observe when 50 % of patients have died. However, with gradual improvements in post-transplant survival, even median survival can exceed 15 years. The survival models for LYFT were developed based on transplant recipient data spanning the period 1987 to 2006, thus highlighting another dilemma: predicting survival times based on data from patients who received transplants as many as 20 years ago may not accurately reflect the current clinical situation and the data often need to be further adjusted to reflect improvements in survival over time. For example, advances in surgical technique, organ preservation technology, immunosuppressive therapy and changes in the age and comorbidity profiles of both donors and recipients all have the potential to influence post-transplant outcomes.

Unlike the Cox regression approach, flexible parametric models characterise the baseline hazard directly and can therefore provide smooth estimates of the hazard and survival functions for any combination of covariates and can be used to extrapolate survival beyond the observed data [6]. The ability to extrapolate also means that it is not necessary to rely on older historical data simply to have sufficient long-term follow-up to observe enough deaths. By choosing to focus on data from transplants that have been carried out more recently, a parametric modelling approach offers the advantage of allowing us to generate predictions of mean patient survival that are more reflective of the characteristics of the current transplant population and of current clinical practice.

In this analysis, we demonstrate the application of the flexible parametric modelling approach proposed by Royston and Parmar [6, 13] to predict mean patient survival among recipients of kidney transplants from deceased donors in the United Kingdom. We begin by describing the dataset and explaining the approach we took to determine how many years of historical data we should use to inform model development. We then present the fitted flexible parametric model and demonstrate agreement between observed and predicted survival. Finally, we use the model to extrapolate beyond the observed data in order to predict mean survival for patients with a given set of characteristics.

## Methods

### Data source

NHS Blood and Transplant is the central authority responsible for managing the UK Transplant Registry, which records mandatory data for kidney transplants performed in all transplant centres across the UK [3]. Anonymised data on all first-time kidney and combined kidney and pancreas transplants performed between 1993 and 2012 were obtained from the registry. Patients <18 years old at the time of transplant, recipients of kidneys from living donors, en bloc and double transplants were excluded from the analysis, as were recipients of kidneys transplanted with organs other than the pancreas.

### Determining how many years of transplant data to include in model development

Kaplan-Meier curves and log-rank tests were used to explore if there was any evidence of notable shifts in mortality rates over the 20-year period that would justify controlling for change over time or potentially restricting the analysis to more recent years of data. Several approaches for dividing the dataset into cohorts based on year of transplant were explored, including 5-year intervals, 10-year intervals and intervals that coincided with changes to the UK national kidney allocation scheme in 1998 and 2006. The list of variables that were routinely recorded in the UK Transplant Registry changed between 1993 and 2012 and so the availability of key variables was also an important consideration in deciding whether to model survival using all of the data or to limit the analysis to a more recent subset. Based on a combination of the above factors, a decision was made to restrict the development of the flexible parametric model to patients who received transplants between 2003 and 2012; however, longer-term data from transplants performed between 1993 and 2002 were used to check the plausibility of extrapolated survival based on the fitted model.

### Explanatory variables

Previous published analyses and prognostic models were reviewed to identify potential factors for inclusion in the development of the model to predict post-transplant patient survival [3, 8, 9]. Recipient factors of interest included age, gender, ethnicity, primary renal diagnosis, pre-emptive transplant, waiting time, kidney only versus combined kidney and pancreas transplant and the calculated reaction frequency of antibodies to human leukocyte antigen (HLA). Calculated reaction frequency (cRF) is a measure of the sensitisation level for each patient and is calculated as the percentage of donors in a pool of 10,000 UK donors with whom the patient is HLA antibody incompatible, similar to the concept of calculated panel reactive antibody [2]. Patients with a cRF between 0 and 9 % were considered non-sensitised, whereas patients with a cRF ≥ 85 % were classed as highly sensitised [14]. Donor factors of interest included age, ethnicity, weight, history of hypertension, diabetes, circulatory-death versus brain-death donor and cause of death. Cold ischaemia time and the level of HLA mismatch were also included. HLA mismatch was graded from level 1 (000-mismatched) to level 4 (poorly matched) as described in the UK 2006 National Kidney Allocation Scheme [15].

Categorical variables were created for each of these factors and the univariate effect of each factor on survival was explored using log-rank tests [16]. After making the decision to restrict model development to patients who received transplants between 2003 and 2012, most variables had either complete or only a small amount of missing data (<2 %) and therefore we did not perform imputation in order to facilitate the model fitting process. However, data for two donor factors, hypertension and diabetes, were missing in approximately 8 % of cases. For these variables, two approaches to handling missing data were explored. First, in order to retain these cases during model fitting, additional categories for missing donor hypertension and donor diabetes status were created. Second, multiple imputation using chained equations was performed and results were compared for consistency with the non-imputed dataset.

### Fitting the multivariable flexible parametric model

We followed the Royston-Parmar approach to fitting a flexible parametric model, in which the baseline distribution is modelled as a restricted cubic spline function of log time [6, 17]. The first step in the development of the prognostic model was to determine the appropriate complexity or number of knots to characterise the baseline spline function and choose a suitable scale (proportional hazards, proportional odds or probit) [6]. We initially fitted models on each of the three scales while varying the number of interior knots from 0 to 4 and inspected the Akaike information criterion (AIC) to determine the optimal fit.

For the multivariable model, the data were then split 2:1 into derivation and validation subsets and variable selection was performed on the derivation dataset using backward elimination and a p-value threshold of 0.10. We tested selectively for clinically plausible interactions and explored the possibility of time-dependent effects for specific covariates if log-log plots suggested any departures from proportionality of hazards over time. We used the model fitted to the derivation subset to predict survival curves in the validation subset and compared these graphically. The final model was then refitted to the combined derivation and validation dataset and results are reported with the index of concordance (c index) as a measure of discrimination. The c index estimates the probability of concordance between predicted and observed outcomes with a value of 0.5 indicating no predictive discrimination and a value of 1.0 indicating perfect separation of patients with different outcomes [18]. The fitted model was then used to extrapolate survival curves for patients with given characteristics in order to generate predictions of mean survival by calculating the area under the curve.

All analyses were conducted in Stata (Version 13, Stata Corp, College Station, Texas, USA). The flexible parametric model was fitted using the *stpm2* command [17].

## Results

### Restricting model development to transplants carried out between 2003 and 2012

**Fig. 1:**
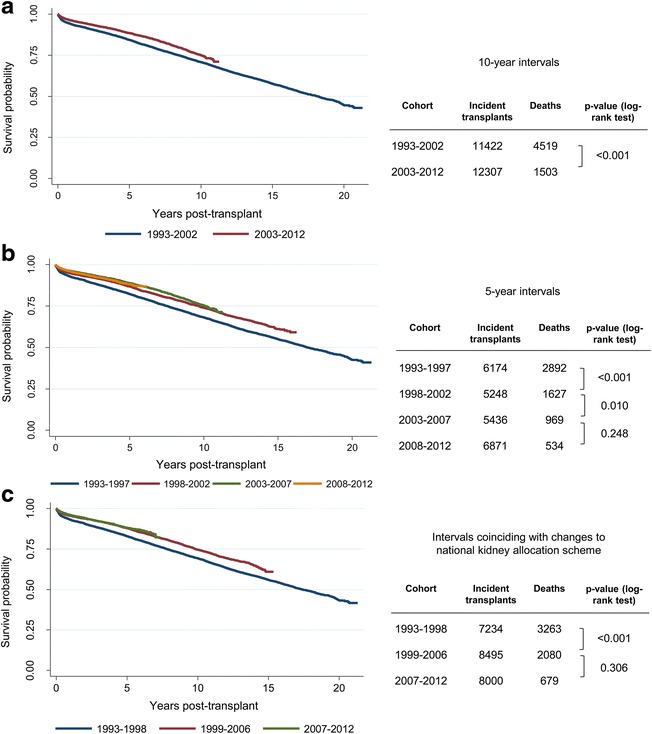
Kaplan-Meier curves and log-rank tests to explore changes in patient survival between different cohorts based on year of transplant: (**a**) 10-year intervals (**b**) 5-year intervals and (**c**) intervals that coincide with changes to the national kidney allocation scheme

The initial dataset included 23,729 patients who received a transplant between 1993 and 2012. Kaplan-Meier curves were plotted for groups defined by year of transplant to explore if there have been any notable shifts in mortality rates over the 20-year period of available data. Visual inspection showed clear separation of survival curves for patients who received a transplant between 1993 and 2002 versus patients who received a transplant between 2003 and 2012, and this difference was confirmed by a log-rank test (Fig. 1a). Alternative approaches to dividing the time period into 5-year intervals (Fig. 1b) or intervals that coincided with changes to the national kidney allocation scheme (Fig. 1c) confirmed that mortality rates did not differ significantly within the last 10 years (between 2003 and 2012) of the dataset; however, improvements in survival were seen when comparing mortality rates within the first 10 years (between 1993 and 2002). In addition to shifts in survival curves, another important consideration for the multivariable analysis was the availability of data for key covariates of interest. For example, data on cold ischaemia time has only been consistently recorded in the registry since 2000 and there were considerable differences in the proportion of circulatory-death donors between the years 1993 and 2002 (3.7 %) and the years 2003 and 2012 (28.4 %). Therefore based on the observed improvements in survival and availability of data, a decision was made to restrict the development of the survival model to those patients who received transplants between 2003 and 2012.

### Univariate analysis

Table 1 summarises the results of univariate survival analyses by recipient, donor and transplant factors. At a *p*-value threshold of 0.05, only three of the factors investigated did not yield statistically significant differences in patient survival: cRF, cold ischaemia time and whether the patient received a kidney only or combined kidney and pancreas transplant.

**Table 1:** Univariate survival analysis by recipient, donor and transplant factors for transplants carried out between 2003 and 2012 (*N* = 12,307)

	n	%	Observed deaths	Crude mortality rate %	*p*-value (log-rank test)
Recipient age					
18-29	997	8.1	43	4.3	<0.001*
30-39	2034	16.5	115	5.7	
40-49	3185	25.9	256	8.0	
50-59	3110	25.3	407	13.1	
> 60	2981	24.2	682	22.9	
Recipient gender					
Male	7628	62.0	984	12.9	0.002
Female	4673	38.0	517	11.1	
Not reported	6	0.1	-	-	
Recipient ethnicity					
White	9871	80.2	1248	12.6	0.033
Asian	1376	11.2	164	11.9	
Other	1049	8.5	90	8.6	
Not reported	11	0.1	-	-	
Transplanted organs					
Kidney only	11013	89.5	1368	12.4	0.253
Kidney and pancreas	1294	10.5	135	10.4	
Pre-emptive transplant					
No	11019	89.5	1406	12.8	<0.001
Yes	1270	10.3	92	7.2	
Not reported	18	0.2	-	-	
cRF					
0-9 %	10026	81.5	1229	12.3	0.356
10-29 %	523	4.3	55	10.5	
30-84 %	1357	11.0	171	12.6	
85-100 %	401	3.3	48	12.0	
Waiting time					
< 6 months	1941	15.8	243	12.5	0.003*
6 months to <2 years	4129	33.6	538	13.0	
> 2 years	6237	50.7	722	11.6	
Primary renal disease					
Glomerulonephritis	1849	15.0	186	10.1	<0.001
Diabetic nephropathy (type 1)	1705	13.9	230	13.5	
Diabetic nephropathy (type 2)	380	3.1	71	18.7	
Renal vascular disease	545	4.4	78	14.3	
Polycystic kidney disease	1513	12.3	147	9.7	
Pyelonephritis	804	6.5	95	11.8	
Other	1573	12.8	181	11.5	
Not reported	3938	32.0	515	13.1	
Donor age					
< 40	3650	29.7	306	8.4	<0.001*
40-49	2754	22.4	324	11.8	
50-59	3200	26.0	416	13.0	
> 60	2703	22.0	457	16.9	
Donor type					
Brain-death donor	8812	71.6	1117	12.7	0.003
Circulatory-death donor	3495	28.4	386	11.0	
Donor hypertension					
No	8688	70.6	938	10.8	<0.001
Yes	2525	20.5	386	15.3	
Not reported	1094	8.9	179	16.4	
Donor diabetes					
Negative	10790	87.7	1268	11.8	0.021
Positive	541	4.4	69	12.8	
Not reported	976	7.9	166	17.0	
Donor weight					
< 55 kg	3150	25.6	380	12.1	0.036
55-65 kg	723	5.9	62	8.6	
65-75 kg	1721	14.0	215	12.5	
75-85 kg	3234	26.3	411	12.7	
85-95 kg	1973	16.0	226	11.5	
> 95 kg	1342	10.9	168	12.5	
Not reported	164	1.3	-	-	
Donor cause of death					
Trauma	1510	12.3	158	10.5	<0.001
Intracranial	7954	64.6	1059	13.3	
Other	2843	23.1	286	10.1	
HLA mismatch					
Level 1 [000]	1485	12.1	193	13.0	0.001*
Level 2 [0 DR + 0/1 B]	4002	32.5	467	11.7	
Level 3 [0 DR + 2 B] or [1 DR + 0/1 B]	5192	42.2	624	12.0	
Level 4 [1 DR + 2 B] or [2 DR]	1628	13.2	219	13.5	
Cold ischaemia time					
< 12 hrs	2061	16.8	177	8.6	0.310*
12 to <18 hrs	5859	47.6	691	11.8	
18 to <24 hrs	2930	23.8	427	14.6	
> = 24 hrs	1264	10.3	186	14.7	
Not reported	193	1.6	-	-	

*log-rank test for trend

### Shape of the hazard function and choice of spline function

Based on AIC, the preliminary flexible parametric model with the optimal fit was found to be on a proportional hazards scale with 2 interior knots for the spline function. Before fitting the multivariable model, we compared the preliminary model based on the chosen scale and number of knots without covariates to the observed data by examining the shape of the hazard and survival functions.

**Fig. 2:**
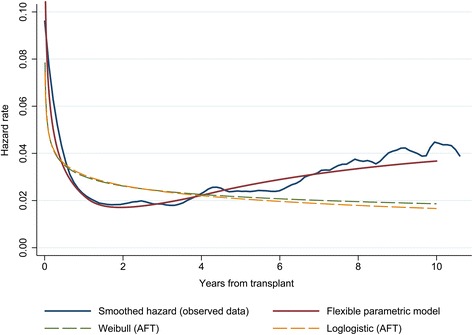
Comparison of smoothed hazard function based on observed data and preliminary flexible parametric model (no covariates) fitted with spline function (2 interior knots); Weibull and loglogistic models in the accelerated-failure time (AFT) metric are also shown for comparison

The risk of death is highest in the period immediately following surgery, then drops sharply before it starts to gradually increase at approximately 2 years post-transplant. Figure 2 demonstrates the ability of the flexible parametric model to accommodate a hazard function that is consistent with the shape of the observed data. This provides reassurance of the improved fit that can be obtained when using splines instead of standard parametric models such as the Weibull or loglogistic shown in Fig. 2 for comparison.

### Fitting the multivariable flexible parametric model

The variable selection process to identify significant predictors of post-transplant survival resulted in the model shown in Table 2. The results in Table 2 reflect the final model fitted to the combined derivation and validation subsets. The risk of death increased with increasing age of both the recipient and the donor, with a primary renal diagnosis of diabetic nephropathy (type 1 or type 2 diabetes) in the recipient and with the presence of hypertension in the donor. The risk of death was lower for female transplant recipients, patients with polycystic kidney disease and patients who received a pre-emptive transplant. Excluding age, type 1 diabetic nephropathy was associated with the highest increase in the risk of death among transplant recipients.

**Table 2:** Final flexible parametric model fitted to combined derivation and validation dataset showing coefficients for each of the 3 spline terms for the baseline hazard function and hazard ratios for significant predictors of post-transplant patient survival (*N* = 12,283)

Baseline hazard (log hazard scale)	Coefficient	*p* **-**value	95 % CI		
Restricted cubic spline 1	1.03	<0.001	0.97	-	1.09
Restricted cubic spline 2	-0.08	0.001	-0.12	-	-0.03
Restricted cubic spline 3	-0.14	<0.001	-0.16	-	-0.12
Constant	-3.97	<0.001	-4.31	-	-3.63
	Hazard ratio	*p*-value	95 % CI		
Recipient age					
18-29	Baseline				
30-39	1.15	0.423	0.81	-	1.64
40-49	1.79	<0.001	1.29	-	2.48
50-59	3.22	<0.001	2.35	-	4.43
> = 60	6.56	<0.001	4.79	-	8.98
Recipient gender					
Male	Baseline				
Female	0.89	0.028	0.80	-	0.99
Pre-emptive transplant					
No	Baseline				
Yes	0.66	<0.001	0.53	-	0.82
Primary renal diagnosis					
Glomerulonephritis	Baseline				
Diabetic nephropathy (type 1)	2.24	<0.001	1.84	-	2.73
Diabetic nephropathy (type 2)	1.59	0.001	1.21	-	2.09
Polycystic kidney disease	0.81	0.056	0.65	-	1.01
Other	1.28	0.007	1.07	-	1.53
Not reported	1.28	0.004	1.08	-	1.52
Donor hypertension					
No	Baseline				
Yes	1.27	<0.001	1.12	-	1.44
Not reported	1.20	0.023	1.03	-	1.42
Donor age					
< 40	Baseline				
40-49	1.26	0.004	1.08	-	1.48
50-59	1.26	0.003	1.08	-	1.47
> = 60	1.48	<0.001	1.26	-	1.74

Interaction terms for recipient age and gender, recipient age and diabetic nephropathy as the primary renal diagnosis, and donor age and hypertension history were tested, but none were found to be significant. To allow for the possibility of time-dependent effects for any of the covariates in the model, we first examined log-log plots for any potential departures from the proportional hazards assumption and identified pre-emptive transplant, type of transplant (kidney only versus combined kidney and pancreas transplant) and cold ischaemia time as potentially varying over time. We tested time-dependent effects for these variables in the flexible parametric model, but again none were found to improve the fit of the model.

### Agreement between observed and predicted survival

**Fig. 3:**
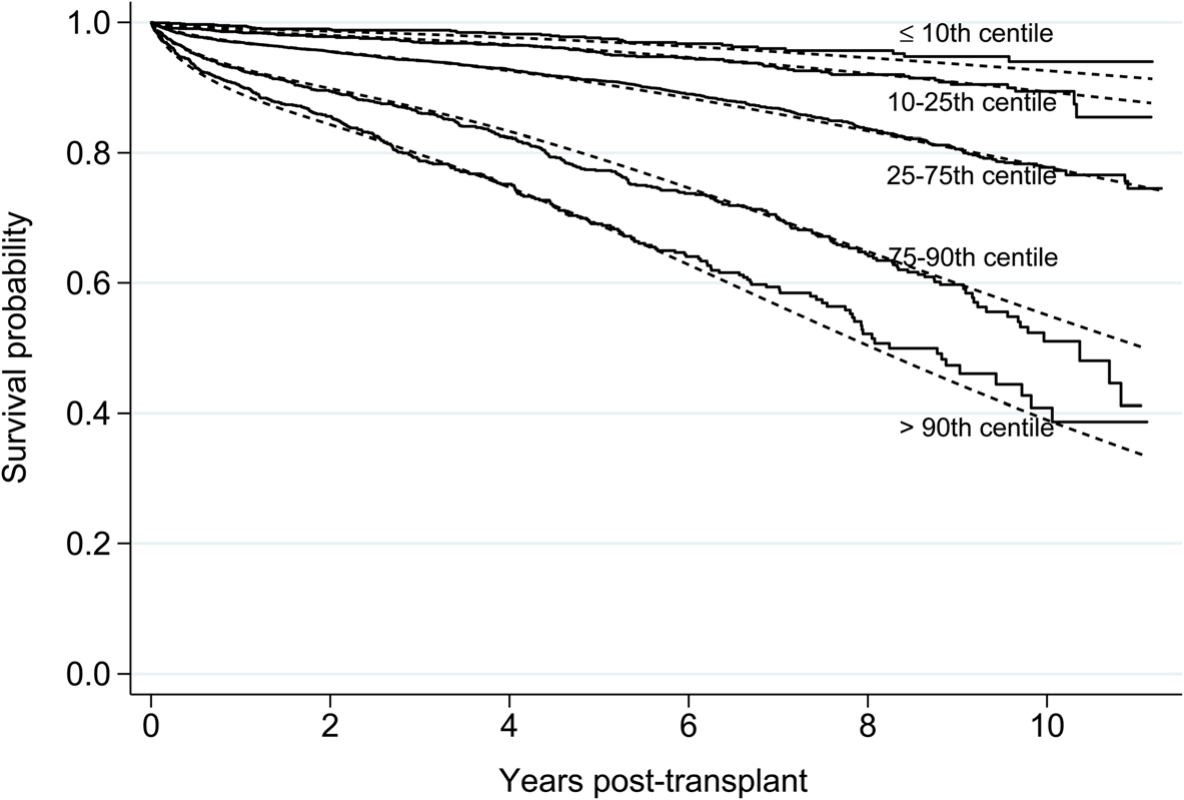
Comparison of Kaplan-Meier curves based on observed data (solid lines) and predicted mean survival curves based on final flexible parametric model (dotted lines) by prognostic group

The c index for the final model was 0.70, comparable to the value reported in the development of the LYFT model (0.68) [19]. To assess the predictive performance of the model, we created five prognostic groups and used the final flexible parametric model to generate a mean survival curve for each group and compared this to the Kaplan-Meier survival curves based on the observed data. Figure 3 shows broad agreement between predicted mean survival curves and the observed Kaplan-Meier curves, although there is less agreement in later years when heavier censoring occurs. The separation of the curves in Fig. 3 also provides insight into the magnitude of survival differences among transplant recipients across the risk spectrum.

### Extrapolation beyond the observed time period to predict mean survival

**Fig. 4:**
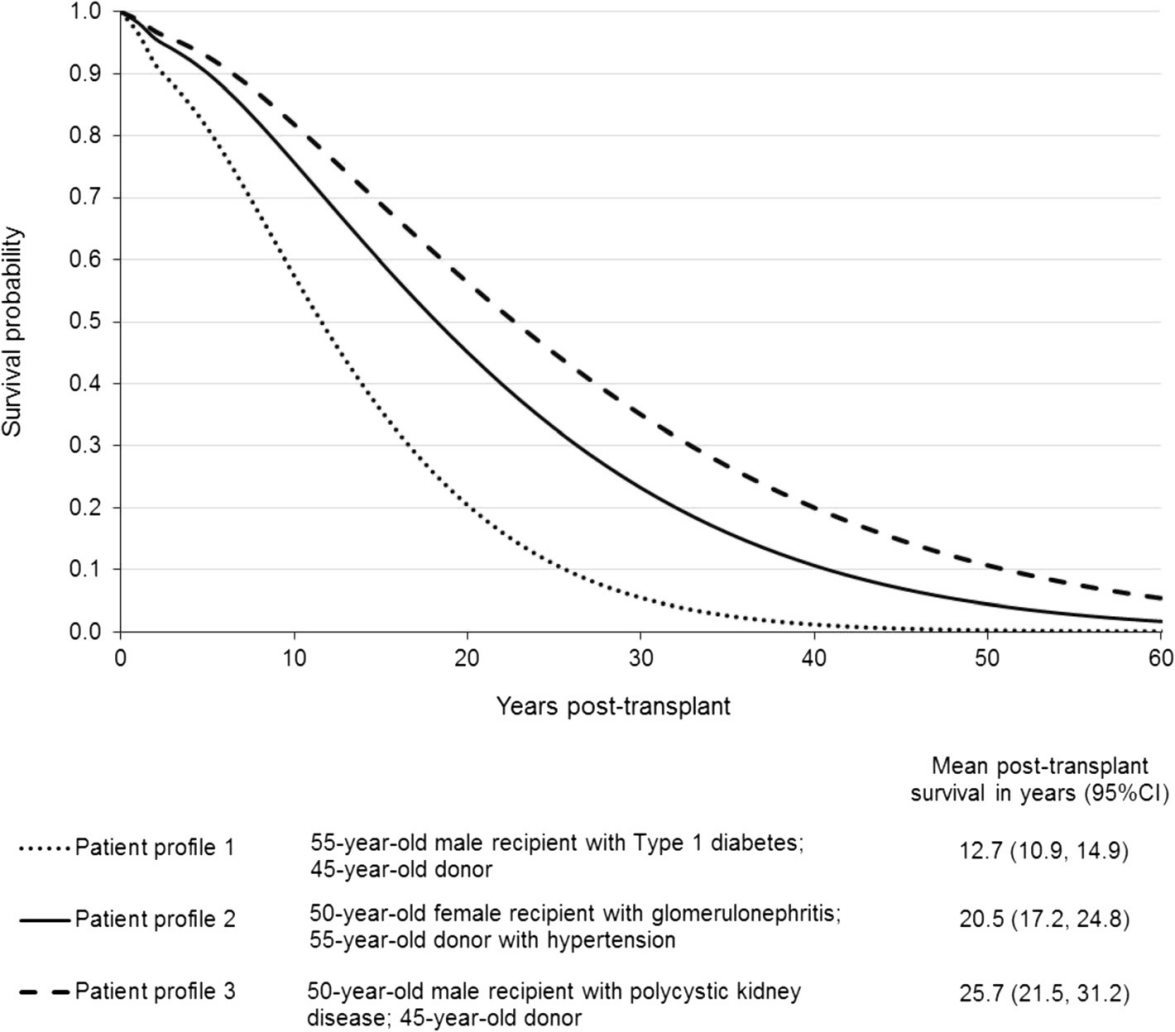
Extrapolated survival curves with mean predicted survival for three different patient profiles

To demonstrate the value of flexible parametric models for extrapolation beyond the period of observed data, we created three hypothetical patient profiles and generated complete survival curves for each of them. Figure 4 shows the differences in survival curves and predicted mean survival by calculating the area under the curve for each patient profile.

## Discussion

There are many examples in the transplant literature of analyses that have examined the influence of various factors on patient survival following kidney transplantation, most of which are based on Cox regression models [3–5]. The objective of the current analysis was to revisit post-transplant mortality using a different modelling technique that facilitates extrapolation of survival curves beyond the period of observed data and allows us to predict mean patient survival times.

Before fitting a flexible parametric model, we felt it was important to first consider how much historical data to include in the development of our model. A conventional approach might be to try and maximise sample size and number of years of follow-up in order to capture any changes in the hazard rate over as long a period as possible; however, we felt that this needed to be balanced with the objective of developing a predictive model that reflects current expectations of post-transplant survival. Although 20 years of historical data on transplants were available for analysis, we chose to restrict model development to the most recent 10 years for two main reasons. First, our exploratory analysis of Kaplan-Meier curves indicated that there had been significant improvements in survival for patients who received transplants between 2003 and 2012 in comparison to patients who received transplants between 1993 and 2002. Second, a wider number of variables that were of potential interest as predictors in the survival model were only available in the more recent subset of the data, including sufficient sample sizes to facilitate a comparison between recipients of organs from circulatory-death donors and brain-death donors. Although restricting model development to transplants performed between 2003 and 2012 reduced the overall sample size and limited the maximum duration of follow-up to 10 years, it was judged that on balance, an analysis based on the more recent subset of data would be a better reflection of current clinical practice and more appropriate given the intended use of the model for predicting survival. Quite often the decision about how much historical data to include in model development is determined primarily by availability of and access to information sources. While the decision that we took to only use the most recent 10 years of transplant data is not widely generalisable beyond our analysis, we advocate considering changes in the clinical context that might influence survival and using exploratory analysis to provide empirical guidance to inform this decision prior to model fitting.

A range of potential explanatory variables were considered during the model development process, but the final model was reduced to just four recipient factors (age, gender, primary renal diagnosis and pre-emptive transplant) and two donor factors (age and hypertension). Notably, we found no difference in death rates between recipients of kidneys from circulatory-death donors in comparison to brain-death donors. In addition, controlling for type 1 diabetic nephropathy as the primary renal diagnosis, we found no difference in death rates for recipients of kidney only transplants compared to recipients of combined kidney and pancreas transplants. These findings are broadly consistent with previous UK analyses based on Cox regression models. For example, Johnson et al identified recipient age, donor age and diabetes to be significant predictors of 5-year patient survival [3]. However, Johnson et al. found that a waiting time of 2 years or more and hypertension as the primary renal diagnosis in transplant recipients also significantly increased the risk of death at 5 years. In the present analysis, hypertension was grouped with other forms of renal vascular disease as a primary diagnosis, the latter which was also not found to be a significant predictor of survival by Johnson et al. The analysis by Johnson et al. was based on a slightly earlier time period and included patients who received transplants in the UK between 1995 and 2001; it did not include recipients of combined kidney and pancreas transplants or recipients of organs from circulatory-death donors. With respect to donor factors, the current analysis reaches similar conclusions to the findings of Watson et al. in the development of the UK Kidney Donor Risk Index (KDRI), which identified donor age group and donor hypertension as the two most important variables with the largest influence on transplant outcomes [20].

The UK Transplant Registry is a rich source of historical data and among patients who received transplants after 2002, many of the variables that we explored in our model had either complete or only small amounts of missing data. However, the amount of missing data for variables such as recipient primary renal diagnosis and donor hypertension potentially introduce an additional source of uncertainty into our final predictive model. For donor hypertension, we performed multiple imputation and confirmed that this did not change the effect of this variable on post-transplant survival estimates. Nonetheless, information on donor hypertension in the registry is obtained from various sources, ranging from medical records to family members, and we were unable to control for consistency with respect to the definition of donor hypertension in the dataset. The prominence of donor hypertension in post-transplant survival models highlights the importance of improving the completeness and consistency with which this variable is recorded. In addition, the registry does not contain information on other factors such as comorbidities or dialysis history for transplant recipients, so we were unable to explore the potential effect of these variables on patient survival in the current analysis.

## Conclusion

The flexible parametric approach to modelling survival offers several advantageous features. In comparison to semi-parametric approaches such as the Cox regression model, fully parametric models characterise the baseline hazard, which facilitates extrapolation beyond the period of observed data. In comparison to standard parametric models such as the Weibull, the use of restricted cubic splines allows for greater flexibility to accommodate more complex hazard functions that increase and decrease over time and are commonly encountered in medical research. The objective of this analysis was to demonstrate the application of a flexible parametric modelling approach to predict mean survival times for recipients of kidney transplants. The application of flexible parametric techniques to estimate mean survival in patients who are receiving dialysis would facilitate comparisons of survival differences between alternative treatment modalities. In addition to informing cost-effectiveness analyses, this approach may have a variety of applications, from the development of prognostic models for informing discussions with patients about treatment outcomes to the use of scoring tools as part of organ allocation schemes. Given the advantages of flexible parametric models, we feel that it is a particularly useful approach for conducting multivariable analysis of patient-level observational data when the goal is to predict long-term survival.

## Abbreviations

AIC, Akaike information criterion; cRF, calculated reaction frequency; EPTS, expected post transplant survival; HLA, human leukocyte antigen; KDPI, kidney donor profile index; KDRI, kidney donor risk index; LYFT, life years from transplant; RRS, recipient risk score.
